# Complete mitochondrial genome of the *Loligo chinensis*

**DOI:** 10.1080/23802359.2017.1361347

**Published:** 2017-08-02

**Authors:** Lihua Jiang, Lisen Kang, Changwen Wu

**Affiliations:** National Engineering Research Center of Marine Facilities Aquaculture, College of Marine Science and Technology, Zhejiang Ocean University, Zhoushan, People’s Republic of China

**Keywords:** Mitochondrial genome, *Loligo chinensis*, phylogenetic tree

## Abstract

In this study, we determined the complete mitochondrial genome of *Loligo chinensis*. The genome was 17,353 bp in length and contained 13 protein-coding genes, 22 transfer RNA genes, 2 ribosomal RNA genes and 3 main non-coding regions. The overall base composition of *L. chinensis* is A 38.62%, C 19.40%, T32.37% and G 9.61%, with a highly A + T bias of 70.99%. All of the three control regions (CR) contain termination-associated sequences and conserved sequence blocks. Here, we describe a phylogenetic analysis of 11 species Cephalopoda based on the complete mitochondrial genome; the result showed that the *Loligo edulis* is most closely related to *L. chinensis.* This mitogenome sequence data would play an important role in the investigation of phylogenetic relationship, taxonomic resolution and phylogeography of the Cephalopoda.

*Loligo chinensis*, which belongs to Loliginidae, Teuthoidea, Coleoidea (Cephalopoda), Animalia (Mollusca) (Dong 1988), is one of the most economically marine cephalopod (Dong 1988). In order to provide useful information for the future research of genetic diversity and phylogenetics, we determined the complete mitochondrial genome of *L. chinensis* (GenBank accession number KT362380).

*L. chinensis* were obtained from the otter trawl. Initially, the squid were identified based on both the morphologic features and the COI mitochondrial gene. Tissue samples for molecular analysis were reserved in 95% ethanol. Whole genomic DNA was extracted from muscle tissue of individual specimens using the phenol–chloroform method. The DNA of the sample is stored in the lab of National Engineering Research Center of Marine Facilities Aquaculture, College of Marine Science, Zhejiang Ocean University, Zhoushan, Zhejiang Province, China. Polymerase chain reaction (PCR) was performed. The PCR products were sequenced by Sangon Biotech (Shanghai, China).

Sequences were assembled using Geneious 4.5.3 (http://www.geneious.com). BioEdit 7.0 (Hall 1999) was used for sequence alignment. The neighbour joining (NJ) methods were used to construct the phylogenetic tree. The NJ trees were obtained with 1000 bootstrap replications using MEGA5.0 (Kimura 1980).

The complete mitochondrial genome of *L. chinensis* is 17,353 bp in length and consists of 13 protein-coding genes, 22 transfer RNA genes (tRNA), 2 ribosomal RNA genes (rRNA) and 3 control regions (CR). The mitogenome base composition was A 38.62%, C 19.40%, T32.37% and G 9.61%, and A + T content (70.99%) was much higher than the G + C content, in common with other invertebrate mitogenomes.

Thirteen protein-coding genes can be classified into two categories: CYTB, ND6, ND1, ND5, ND4 and ND4L were encoded by the light-strand, and the rests including COIII, ND3, ND2, COI, COII, ATP8 and ATP6 were encoded by the heavy strand. Seven protein-coding genes (COX3, ND3, ND2, COX1.COX2, ATP8 and ATP6) start with an ATG initiation codon, ND1 and ND6 start with an TTA initiation codon, and CYTB and ND4 share CTA as the initiation codon, while ND5 uses CAT as the initiation codon, and ND4L uses ATA as the initiation codon. Six protein-coding genes (COX3, ND2, COX1, COX2, ND3 and ATP6) use TAA as the termination codon; five protein-coding genes (Cytb, ND6, ND1, ND5, ND4L) use CAT as the termination codon, while ND4 uses TAT as the termination codon; ATP8 uses TAG as the termination codon. The two ribosomal RNA genes, 16SrRNA (1334 bp) is located between tRNA^Ile^ and tRNA^Val^ genes, and 12SrRNA (1006 bp) is located between tRNA^Val^ and tRNA^Trp^ genes. The complete mitochondrial genome of *L. chinensis* has three non-coding regions, and they were 514 bp, 535 bp and 518 bp, respectively, in length and contain termination-associated sequences and conserved sequence blocks: one was located between tRNA^Gln^ and tRNA^Ile^, one was located between tRNA^Trp^ and tRNA^Lys^, and the other one was located between tRNA^Gly^ and tRNA^Ala^.

In this study, *Loligo edulis* is most closely related to *L. chinensis*, *Octopus ocellatus* was placed at the most basal position forming an individual clade, while other species formed another large cluster. *Loligo opalescence* was grouped with *Loligo duvaucelii*, *Loligo uyii* and *Loligo beka* (Figure 1).

**Figure 1.**
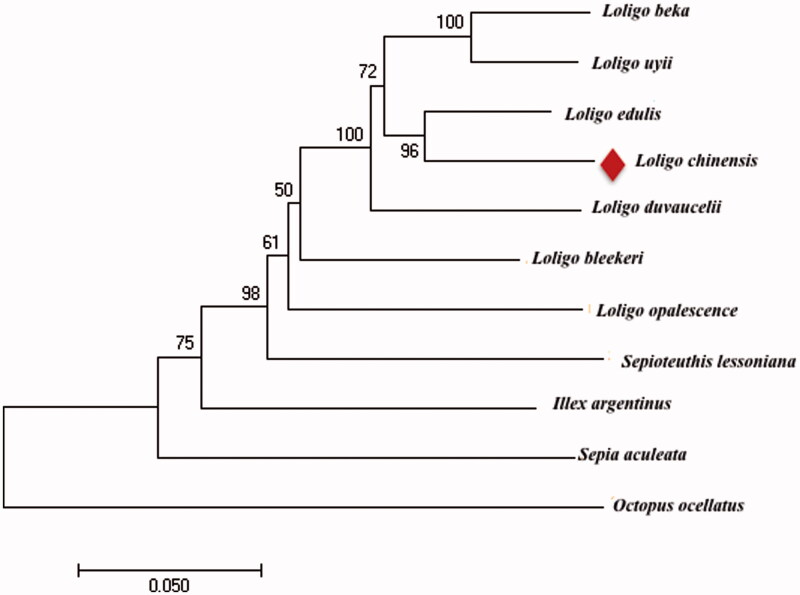
Molecular phylogenetic tree based on the complete mitochondrial genome sequences. GenBank accession numbers: *Loligo beka*: KT254309, *Loliolus uyii*: NC026724, *Loligo edulis*: AB675081, *L. chinensis*: KT362380, *Loligo bleekeri*: NC002507, *Sepioteuthis lessoniana*: AB240154, *Loligo duvaucelii*: KR051264, *Loligo opalescence*: KP336703, *Illex argentinus*: KP336702, *Todarodes pacificus*: NC006354, *Sepia aculeata*: KF690633 and *Octopus ocellatus*: NC007896.

*L. chinensis* is one of the economically marine cephalopod. We expect that the present result will facilitate the further investigations of phylogenetic relationship, taxonomic resolution and phylogeography of the cephalopod.

## References

[CIT0001] Dong ZZ. 1988 Chinese Fauna, Mollusca, Cephalopods. Beijing: Science Press.

[CIT0002] Hall TA. 1999 BioEdit: a user-friendly biological sequence alignment editor and analysis program for Windows 95/98/NT. Nucleic Acids Symp Ser. 41:95–98.

[CIT0003] Kimura M. 1980 A simple method for estimating evolutionary rates of base substitutions through comparative studies of nucleotide sequences. J Mol Evol. 16:111–120. 7463489 10.1007/BF01731581

